# Effects of Race and Gender Classifications on Atherosclerotic Cardiovascular Disease Risk Estimates for Clinical Decision-Making in a Cohort of Black Transgender Women

**DOI:** 10.1089/heq.2023.0066

**Published:** 2023-11-30

**Authors:** Tonia Poteat, Elle Lett, Ashleigh J. Rich, Huijun Jiang, Andrea L. Wirtz, Asa Radix, Sari L. Reisner, Alexander B. Harris, Jowanna Malone, William G. La Cava, Catherine R. Lesko, Kenneth H. Mayer, Carl G. Streed

**Affiliations:** ^1^ Department of Social Medicine, University of North Carolina School of Medicine, Chapel Hill, North Carolina, USA.; ^2^ Computational Health Informatics Program, Boston Children's Hospital, Boston, Massachusetts, USA.; ^3^ Perelman School of Medicine, University of Pennsylvania, Philadelphia, Pennsylvania, USA.; ^4^ Center for Applied Transgender Studies, Chicago, Illinois, USA.; ^5^ Department of Biostatistics, Gillings School of Public Health, University of North Carolina, Chapel Hill, North Carolina, USA.; ^6^ Department of Epidemiology, Johns Hopkins Bloomberg School of Public Health, Baltimore, Maryland, USA.; ^7^ Callen Lorde Community Health Center, New York, New York, USA.; ^8^ Division of Endocrinology, Diabetes and Hypertension, Department of Medicine, Brigham Women's Hospital, Boston, Massachusetts, USA.; ^9^ Department of Medicine, Harvard Medical School, Boston, Massachusetts, USA.; ^10^ Department of Epidemiology, Harvard School of Public Health, Boston, Massachusetts, USA.; ^11^ The Fenway Institute, Fenway Health, Boston, Massachusetts, USA.; ^12^ Whitman Walker Institute, Washington, District of Columbia, USA.; ^13^ Department of Pediatrics, Harvard Medical School, Boston, Massachusetts, USA.; ^14^ Department of Medicine, Beth Israel Deaconess Medical Center, Boston, Massachusetts, USA.; ^15^ Section of General Internal, Department of Medicine, Boston University Chobanian and Avedisian School of Medicine, Boston, Massachusetts, USA.; ^16^ GenderCare Center, Boston Medical Center, Boston, Massachusetts, USA.

**Keywords:** transgender, African American, cardiovascular risk calculator, statin

## Abstract

**Introduction:**

Despite their dynamic, socially constructed, and imprecise nature, both race and gender are included in common risk calculators used for clinical decision-making about statin therapy for atherosclerotic cardiovascular disease (ASCVD) prevention.

**Methods and Materials:**

We assessed the effect of manipulating six different race-gender categories on ASCVD risk scores among 90 Black transgender women.

**Results:**

Risk scores varied by operationalization of race and gender and affected the proportion for whom statins were recommended.

**Discussion:**

Race and gender are social constructs underpinning racialized and gendered health inequities. Their rote use in ASCVD risk calculators may reinforce and perpetuate existing inequities.

## Introduction

Atherosclerotic cardiovascular disease (ASCVD) is the leading cause of morbidity and mortality in the United States.^1^ Black adults are more likely to have ASCVD risk factors and more than twice as likely to die from it, compared to White adults.^2^ Although ASCVD incidence among cisgender women is lower than men, evidence suggests that cisgender women have higher mortality and poorer prognosis following an acute cardiovascular event compared with cisgender men.^3^

Unfortunately, most studies of gender differences in ASCVD conflate gender and sex, focus largely on physiological differences between males and females, and fail to consider transgender people.^4^ Data on ASCVD among transgender populations are limited.^5^ However, an analysis of nationally representative data found transgender people had higher odds of self-reported heart attacks compared with cisgender people.^6^ A more recent study found transgender women had worse cardiovascular biomarker profiles than cisgender men independent of hormone use and HIV serostatus.^7^

Statin therapy is highly effective in reducing risk of ASCVD events and is recommended by national clinical guidelines for individuals at elevated risk.^8^ However, treatment with statins has been associated with risk of adverse events including myopathy, liver dysfunction, renal insufficiency, and diabetes.^9^ Accurately predicting the risk for ASCVD events is essential for clinical decision-making about the risks versus benefits of statin therapy for prevention.^8^

Statistical algorithms, such as the American College of Cardiology (ACC)/American Heart Association (AHA) ASCVD Risk Calculator, are commonly used to facilitate efficient, noninvasive, and potentially life-saving clinical decisions.^10^ This algorithm uses clinical data such as lipid levels and blood pressure as well as social categories, race, and gender, to calculate risk of an ASCVD event over the next 10 years. ACC/AHA guidelines recommend statin therapy initiation if the calculated risk meets or exceeds 7.5%. When using the risk calculator, options for race include White, African American, and Other; however, the algorithm assigns the same weight to White and Other. Options for gender include male and female.

Intersectionality, as a theoretical framework and critical praxis, explicates the ways in which social categories, at the individual level, reflect interlocking systems of power and oppression at the structural level.^11^ Race and gender are particularly salient categories typically assessed in medicine as if they are fixed and exclusively biological constructs. However, these categories are socially constructed, multidimensional, and have changeable borders that are dynamic across time as well as within and across persons.^12,13^ One's social location at the intersection of race and gender fundamentally impacts one's lived experiences and therefore health.^14–16^

Black transgender women, who experience multiple marginalization along axes of race and gender, experience health inequities rooted in racism and transmisogyny.^5,17^ However, they are rendered invisible in medical education and nearly all available clinical algorithms.^18–21^ Published medical literature has begun to challenge the use of race in clinical algorithms.^22^ However, we found no studies that challenged the use of both race and gender in clinical algorithms nor examined the impact of their combined use in clinical decision-making.

In this analysis, we assess the effects of embedding these intersecting social categories in a clinical algorithm that shapes medical decision-making.

## Materials and Methods

We conducted a cross-sectional analysis of baseline data collected between October 2020 and June 2022 from 90 Black transgender women enrolled in LITE Plus, an ongoing, longitudinal study of chronic stress, allostatic load, and ASCVD among Black and Latina transgender women living with HIV.^23^ For the parent study, participants were recruited from health centers in Boston, New York, and Washington, DC. Eligibility included age 18 years or older, male sex assigned at birth, current gender identity along a trans feminine spectrum, self-reported race as Black or ethnicity as Latina, and laboratory-confirmed HIV. The University of North Carolina Institutional Review Board provided ethical review and approval (No. 18-2632). We restricted this analysis to the 90 participants who identified as Black, regardless of ethnicity or additional racial identities.

Self-reported measures included smoking status (never, former, current), current medications, and diagnosed health conditions. Where possible, medication lists and diagnosed health conditions were corroborated by medical record review. Anthropomorphic measures included systolic and diastolic brachial blood pressure. Laboratory tests included glycosylated hemoglobin and fasting total cholesterol, high-density lipoprotein cholesterol (HDL), and low-density lipoprotein cholesterol (LDL).

Data analyses were conducted using R Statistical Software (version 4.2.1; R Core Team 2022). We generated ASCVD risk scores using the *CVrisk* package in R based on the ACC/AHA ASCVD Risk Calculator algorithm.^24^ We excluded participants for whom the ASCVD risk calculator was invalid: seven participants with a history of heart disease or stroke and five individuals with cholesterol, HDL, or LDL values outside of allowable ranges. Participant risk scores were assessed using three approaches for operationalizing gender/sex: (1) participants' sex assigned at birth (male); (2) current identity (female); and (3) selecting female for participants who were currently taking gender-affirming hormone therapy (GAHT) and selecting male for participant who were not taking GAHT. Per the response options available on the risk calculator, participant scores were assessed designating race as Black or recategorizing race as White. This led to a total of six possible race-gender categorizations used to calculate ASCVD risk scores.

The proportion of participants who met criteria for statin therapy (risk score ≥7.5%) was calculated for each of the six potential race-gender categories. We calculated mean absolute percent differences for each potential pairing. To determine the reclassification rate of participants who changed from indicated to not indicated (or vice versa) for a statin when their race-gender categories were changed, we calculated the percent of individuals with different indications between the two calculations for each race-gender category pairing.

## Results

More than half of participants (53.3%) were aged 40 years and 80% were taking GAHT. Dyslipidemia was common: 45.6% had an elevated LDL. Eleven percent had a diabetes diagnosis, 31.1% were current smokers, and 18% were taking a statin. Almost 8% had a history of heart disease or stroke (Table 1).

**Table 1.** Baseline Participant Characteristics of Black LITE Plus Participants, *n* (%) Unless Otherwise Specified

Demographics/clinical characteristics	***N*** =90
Age in years, mean (range)	43 (21–71)
≥40 years old	48 (53.3)
Currently taking gender-affirming hormones	72 (80.0)

ASCVD risk factors	***N*** =90
Systolic blood pressure, mean	126 (14.0)
≥140 mm Hg	15 (16.7)
Diastolic blood pressure, mean	80 (9.0)
≥90 mm Hg	10 (11.1)
On antihypertension med	21 (23.3)
Total cholesterol, mean	173 (37.0)
≥200 mg/dL	20 (22.2)
HDL, mean	54 (17.0)
≤40 mg/dL	15 (16.7)
LDL, mean	98 (32.0)
≥100 mg/dL	41 (45.6)
Diabetes	10 (11.1)
% glycosylated hemoglobin ≥6.5	8 (9.0)
Smoking status	
Current smoker	28 (31.1)
Former smoker	23 (25.6)
Never smoker	39 (43.3)
On statin	16 (18.0)
On aspirin	9 (9.0)
History of heart disease (heart failure or myocardial infarction), or stroke	7 (7.8)

All data were complete except % glycosylated hemoglobin and self-reported statin usage, which was missing for one respondent each.

ASCVD, atherosclerotic cardiovascular disease; HDL, high-density lipoprotein cholesterol; LDL, low-density lipoprotein cholesterol.

ASCVD risk scores were lower for White race regardless of gender operationalization, and a lower proportion met the risk threshold for recommending statin therapy. The proportion of participants for whom statin therapy would be recommended was highest when using sex assigned at birth and lowest when using current gender identity. The ASCVD calculator produced the highest mean risk score (6.6%) when participants were assigned both Black and male, with 30.8% meeting criteria for statin therapy. The lowest mean risk score (5.6%) was produced when participants were assigned White and female, with 14% meeting criteria for statin therapy (Table 2).

**Table 2.** Estimated Atherosclerotic Cardiovascular Disease 10-Year Risk Scores Based on Race and Gender Categorization Among Black LITE Plus Participants, *N*=78

Sex variable	Black race (as self-identified)		White race (hypothetical scenario)	
	Mean % (SD)	≥7.5%, ***n*** (%)	Mean % (SD)	≥7.5%, ***n*** (%)
Gender identity (female)	4.44 (5.6)	16 (20.5)	3.22 (3.6)	11 (14.1)
Sex assigned at birth (male)	6.56 (6.5)	24 (30.8)	4.70 (6.0)	16 (20.5)
Female if currently on hormones	5.09 (6.5)	18 (23.1)	3.86 (4.9)	13 (16.7)

Seven participants had history of heart disease (myocardial infarction or heart failure), or stroke. ASCVD calculator is not valid for individuals with a history of myocardial infarction or stroke, so they were excluded. An additional five individuals had cholesterol, HDL, or LDL values outside of the values for which the ASCVD risk calculator is valid.

SD, standard deviation.

The highest mean absolute percent difference in risk scores (69%) and reclassification rate (21.2%) was between assigning participants as Black males versus White females. The lowest mean absolute percent difference (8.3%) and reclassification rate (2.4%) were between assigning participants as White females if on GAHT and assigning them as White females based on gender identity (Table 3).

**Table 3.** Mean Absolute Percent Difference and Reclassification Rates in Atherosclerotic Cardiovascular Disease 10-Year Risk Scores Between Race-Gender Combinations Used for Risk Calculations Among Black LITE Plus Participants, *N*=78

	Black			White		
	GI (female)	SAAB (male)	GAHT	GI (female)	SAAB (male)	GAHT
Mean absolute percent difference (%)						
Black						
GI (female)	—					
SAAB (male)	57.7	—				
GAHT	10.9	46.8	—			
White						
GI (female)	33.0	69.0	42.3	—		
SAAB (male)	25.9	48.4	28.4	30.9	—	
GAHT	32.6	61.8	35.2	8.3	22.6	—
Reclassification rate (%)						
Black						
GI (female)	—					
SAAB (male)	12.9	—				
GAHT	3.5	9.4	—			
White						
GI (female)	10.6	21.2	14.1	—		
SAAB (male)	11.8	10.6	10.6	10.6	—	
GAHT	12.9	18.8	11.8	2.4	8.2	—

Seven participants had history of heart disease (myocardial infarction or heart failure), or stroke. ASCVD calculator is not valid for individuals with a history of myocardial infarction or stroke, so they were excluded. An additional five individuals had cholesterol, HDL, or LDL values outside of the values for which the ASCVD risk calculator is valid.

GAHT, gender-affirming hormone therapy (treated as female if current); GI, gender identity; SAAB, sex assigned at birth.

## Discussion

Among Black transgender women with HIV, ASCVD risk scores varied substantially by how race and gender were operationalized, producing clinically relevant differences in statin therapy recommendations according to ACC/AHA guidelines. Use of the risk calculator requires clinicians not only to assign social categories based on perception or patient report but also to select between binary options—despite the multidimensionality of both race and gender.^4^

The inclusion of race and gender variables in clinical algorithms reflects observed differences in ASCVD rates. However, it also incorrectly assumes race and gender categories are biological and fixed. Drivers of ASCVD inequities among Black Americans are rooted in systemic racism, which increases physiological dysregulation (e.g., allostatic load), increases exposure to ASCVD risk factors (e.g., food apartheid), and decreases access to health care.^25^ If race operates in the algorithm as a proxy for the impact of systemic racism, its inclusion may perpetuate racism by ensuring important clinical decisions will be based on race.^20,22^ At minimum, algorithm-based pharmaceutical interventions fail to address the root causes of ASCVD racial inequities.

Likewise, the inclusion of gender in the calculator assumes a shared biology by people of the same gender. However, transgender and intersex people may not share the same hormonal milieu and physical anatomy as their cisgender and endosex peers. Among cisgender people, hormone levels and physiology are influenced by gonadal function that varies by stage of life, surgical history, and use of exogenous hormones (e.g., contraceptives). If gender operates in the algorithm as a proxy for exposure to subordinate gender roles, its inclusion fails to address root causes of sex and gender differences in ASCVD.

An intersectionality framework calls on us to address the interaction of racism, sexism, and transphobia in the development, implementation, and consequences of race- and gender-based clinical algorithms. Race and gender categories are multidimensional, dynamic, and socially constructed structural factors that drive racialized and gender health inequities. Clinical reliance on current calculator's risks over or under-prescribing and perpetuating clinician biases that affect ASCVD treatment—ultimately reinforcing and perpetuating existing population-level inequities. While several alternative ASCVD risk algorithms are in development, all currently rely on race and gender categories for calculation. Rather than rely on flawed calculators, clinicians should consider using multidimensional frameworks such as Life's Essential Eight^26^ to initiate conversations about cardiovascular health in the context of holistic health parameters.

## Abbreviations Used

ACC: American College of Cardiology
AHA: American Heart Association
ASCVD: atherosclerotic cardiovascular disease
GAHT: gender-affirming hormone therapy
GI: gender identity
HDL: high-density lipoprotein cholesterol
LDL: low-density lipoprotein cholesterol
SAAB: sex assigned at birth
SD: standard deviation
